# Increased Expression of the Auxiliary β_2_-subunit of Ventricular L-type Ca^2+^ Channels Leads to Single-Channel Activity Characteristic of Heart Failure

**DOI:** 10.1371/journal.pone.0000292

**Published:** 2007-03-14

**Authors:** Roger Hullin, Jan Matthes, Sibylle von Vietinghoff, Ilona Bodi, Marta Rubio, Karen D'Souza, Ismail Friedrich Khan, Dennis Rottländer, Uta C. Hoppe, Paul Mohacsi, Eva Schmitteckert, Ralf Gilsbach, Moritz Bünemann, Lutz Hein, Arnold Schwartz, Stefan Herzig

**Affiliations:** 1 Department of Cardiology, Swiss Heart Center Bern, University Hospital, Bern, Switzerland; 2 Department of Pharmacology, University of Cologne, Cologne, Germany; 3 Department of Pharmacology, University of Wuerzburg, Wuerzburg, Germany; 4 Franz Volhard Clinic, Nephrology/Hypertension Section, Medical Faculty of the Charité, Berlin, Germany; 5 University of Cincinnati College of Medicine, Institute of Molecular Pharmacology and Biophysics, University of Cincinnati, Cincinnati, Ohio, United States of America; 6 Center of Molecular Medicine, University of Cologne, Cologne, Germany; 7 Department of Experimental and Clinical Pharmacology and Toxicology, University of Freiburg, Freiburg, Germany; Emory University, United States of America

## Abstract

**Background:**

Increased activity of single ventricular L-type Ca^2+^-channels (L-VDCC) is a hallmark in human heart failure. Recent findings suggest differential modulation by several auxiliary β-subunits as a possible explanation.

**Methods and Results:**

By molecular and functional analyses of human and murine ventricles, we find that enhanced L-VDCC activity is accompanied by altered expression pattern of auxiliary L-VDCC β-subunit gene products. In HEK293-cells we show differential modulation of single L-VDCC activity by coexpression of several human cardiac β-subunits: Unlike β_1_ or β_3_ isoforms, β_2a_ and β_2b_ induce a high-activity channel behavior typical of failing myocytes. In accordance, β_2_-subunit mRNA and protein are up-regulated in failing human myocardium. In a model of heart failure we find that mice overexpressing the human cardiac Ca_V_1.2 also reveal increased single-channel activity and sarcolemmal β_2_ expression when entering into the maladaptive stage of heart failure. Interestingly, these animals, when still young and non-failing (“Adaptive Phase”), reveal the opposite phenotype, ***viz*** *:* reduced single-channel activity accompanied by lowered β_2_ expression. Additional evidence for the cause-effect relationship between β_2_-subunit expression and single L-VDCC activity is provided by newly engineered, double-transgenic mice bearing both constitutive Ca_V_1.2 and inducible β_2_ cardiac overexpression. Here in non-failing hearts induction of β_2_-subunit overexpression mimicked the increase of single L-VDCC activity observed in murine and human chronic heart failure.

**Conclusions:**

Our study presents evidence of the pathobiochemical relevance of β_2_-subunits for the electrophysiological phenotype of cardiac L-VDCC and thus provides an explanation for the single L-VDCC gating observed in human and murine heart failure.

## Introduction

Homeostasis of intracellular Ca^2+^ concentration [Ca^2+^]_i_ is essential for cardiac function and integrity; its dysregulation is a hallmark of advanced heart failure [1], [2]. Voltage-dependent L-type Ca^2+^-channels (L-VDCCs) are the source of trigger Ca^2+^ entering cardiomyocytes [3]. Data derived from numerous studies support an involvement of L-VDCC in pathological changes of [Ca^2+^]_i_ in heart failure. Although still controversial, L-VDCC current density appears unchanged in failing cardiomyocytes [1], [4], [5]. Whole-cell currents are determined by a number of parameters, including number of channels, single-channel current amplitude and time spent in the open state. Therefore, altered number of active channels or activity of individual L-VDCC is not necessarily reflected by calcium current density. In fact, despite no change in whole-cell L-VDCC density (*I_Ca_*), single-channel activity was significantly increased in ventricular myocytes from human end-stage failing hearts [6]. Chen et al. [7] showed attenuated *I_Ca_* increase by (S)-BayK8644 in human failing myocardium whereas basal whole-cell currents were unchanged, indicating that single-channel activity is enhanced while channel density is lowered. These findings confirm the idea of an “electrophysiological heart-failure phenotype” of single L-VDCCs. The biochemical nature of this change in phenotype has not been delineated, although phosphorylation [8], [9] and dephosphorylation [10], [11] have been implicated. Activities of kinases and phosphatases not only change channel function but interfere with neurohumoral modulation of the L-VDCC; ***e.g.*** β-adrenergic regulation is blunted in heart failure possibly due to hyperphosphorylation of L-VDCCs [6], [7]. Using heterologous recombination we have shown that distinct subunit compositions of L-VDCC induce single-channel characteristics similar to the biophysical phenotype of “hyperphosphorylated” L-VDCC [12]. The latter suggests that changes in gene expression of L-VDCC subunits may form the basis of a heart-failure phenotype of L-VDCC. In mammalian hearts L-VDCCs are composed of an ion conducting pore (Ca_V_1.2 or α_1C_), and two auxiliary subunits, an α_2_δ and a β-subunit. Most investigators agree that β-subunit diversity is of physiological and pathophysiological importance [13]–[18]. In fact, some studies have revealed altered β-subunit patterns in human heart failure [19], [20], suggesting that an altered β-subunit expression pattern is of functional relevance. Delineation of pathophysiological mechanisms in human heart is difficult because of wide inter-individual variance, including age, medication, state of disease ***etc.*** Human tissue also offers a limited choice of truly independent variables, such as time, disease stage and treatment options. Animal models offer control of any relevant factor to test pathophysiological concepts. We analyzed β-subunit gene expression in both human non-failing and failing hearts as well as in transgenic mice overexpressing the human Ca_V_1.2 (α_1C_) subunit (tg Ca_V_1.2). The latter was chosen because of phenotypical characteristics common with human heart failure, ***e.g.*** early blunting of β-adrenergic signaling, slow progression towards hypertrophy and calcium overload in failing myocytes [21], [22]. Most importantly, in young (non-failing; “Adaptive State”) tg Ca_V_1.2 mice we previously found concordance of lowered β_2_-subunit expression and decreased activity of single L-VDCC [16]. In the present study we find an increase of single L-VDCC activity accompanied by enhanced expression of β_2_-subunits when these mice have entered the failing state (“Maladaptive State” ≥9 months of age). By examination of a new, double-transgenic mouse bearing both constitutive Ca_V_1.2 and inducible β_2_-subunit overexpression in the heart we show a relationship between subunit expression and channel function.

## Results

### Gating parameters of single L-VDCC in failing human and transgenic myocardium

Single-channel activity of old (≥9 months, and in heart failure) tg Ca_V_1.2 was significantly increased compared to young (4 months, no hypertrophy or heart failure) tg Ca_V_1.2 [16] (Table 1). Henceforth, these animals are referred to as “young” and “old” tg Ca_V_1.2, respectively. Peak ensemble average current (*I*_peak_) in old tg Ca_V_1.2 mice was enhanced (−56±14 fA vs. −23±7 fA, p<0.05) due to an increased fraction of active sweeps, mean open time, and mean open probability, and a decrease of mean closed time (t_closed_). Of further interest, the changes of peak current, fraction of active sweeps and open probability mirror findings obtained from single L-VDCC measurements in human cardiomyocytes from non-failing or failing idiopathic dilated cardiomyopathy (DCM) hearts, respectively [6] (Table 1).

**Table 1:** Single L-VDCC gating of young and old tg Ca_V_1.2 resembles data obtained from human non-failing and failing ventricle

gating parameter	tg Ca_V_1.2	tg Ca_V_1.2	human LV	human LV
	4 months	≥9 months	non-failing	failing (DCM)
peak current I_peak_ [fA]	−23±7	−56±14*	−13±5	−28±5*
fraction of active sweeps f_active_ [%]	53.7±5.3	71.7±8.2(*)	26.4±5.3	56.7±8.0*
open probability P_open _[%]	4.4±1.2	6.4±0.9†	3.2±1.3	6.1±1.6†
mean open time t_open_ [ms]	0.34±0.03	0.45±0.04*	0.54±0.05	0.65±0.04
mean closed time t_closed_ [ms]	6.6±1.0	3.1±0.7*	9.3±1.6	7.5±1.7
number of experiments	13 (13)	11 (5)	16 (12)	9 (6)

In a previous study [6] we found single-channel activity to be significantly increased in ventricular myocytes from human hearts failing due to idiopathic dilated cardiomyopathy compared to non-failing ventricles. In excellent agreement the present study reveals activity of single L-VDCC from ≥9 months old, i.e. failing murine hearts overexpressing the human Ca_V_1.2 to be significantly increased compared to single-channel activity in 4 months old, ***i.e.*** non-failing young transgenics obtained in a previous study [16]. Charge carrier: 70 mM Ba^2+^; holding potential: −100 mV; test potential: +20 mV. Note that Schroder et al. [6] did not use a depolarizing bath solution, thus potentials are approximate values.

* p<0.05 and ^(^*^)^p = 0.07 in a Student's t-test; ^†^p<0.05 in a Mann-Whitney test (performed when data failed normality test). Numbers of experiments given in parentheses indicate number of experiments with only one channel in the patch.

### L-VDCC subunit expression in non-failing and failing human hearts

**Figure 1:**
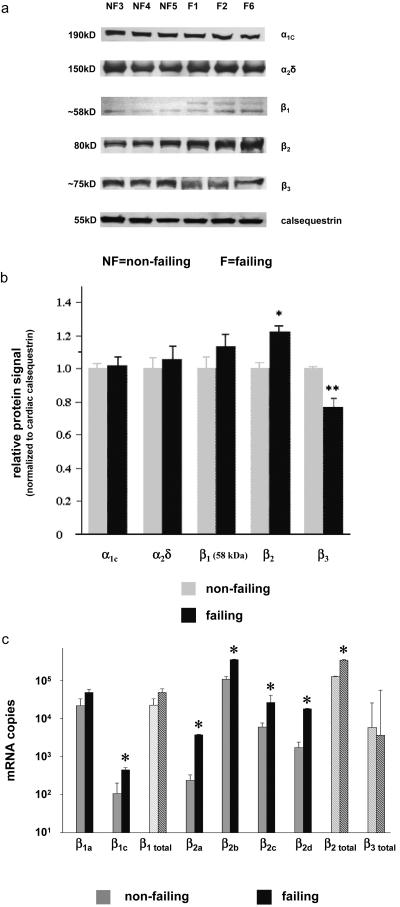
Subunit expression of cardiac L-VDCC subunits in human myocardial specimens **(a)** Human specimens from non-failing (NF) and failing (F) myocardium (n = 4–5) were analyzed in immunoblots using specific polyclonal antibodies directed against the particular L-VDCC subunits. **(b)** L-VDCC subunit expression was normalized to cardiac calsequestrin protein expression in the same sample (number of NF/F specimens was always identical for each subunit; n = 5–8). Quantitative analysis of subunit protein expression is depicted as ratio of F vs. NF. * p<0.001; ** p<0.0001. **(c)** mRNA expression of β-subunit isoforms (NF: n = 5; F: n = 9–13) was measured by real time PCR, and always normalized to cardiac calsequestrin mRNA expression. * p<0.05.

Protein expression of Ca_V_1.2, α_2_δ, low molecular weight β_1_, and β_3_ was similar in non-failing and failing human myocardium, but we found a significant up-regulation of β_2_ (Figure 1a,b). There was no difference in gene expression of the Ca_V_1.2, and the α_2_δ at the protein level (mRNA data not shown). At least two β_1_-subunit isoforms (β_1a,c_), four β_2_-subunit isoforms (β_2a–d_), and two β_3_-subunit isoforms (β_3a,trunc_) are expressed at relevant levels in human myocardium [12]. β_1a_ (GenBank No NM_199247) and β_1c_ (GenBank_199248) are sequence-identical except for replacement of exon 7a by exon 7b in β_1c_, consistent with previous work [23]. β_2a–d_ isoforms differ only with respect to the N-terminal region (D1 domain). Quantitation by real-time PCR revealed an increased expression of β_1c_ and all β_2_ isoforms in heart failure, in line with the protein data (Figure 1c).

### L-VDCC subunit protein expression in old wild type and old tg Ca_V_1.2 hearts

**Figure 2:**
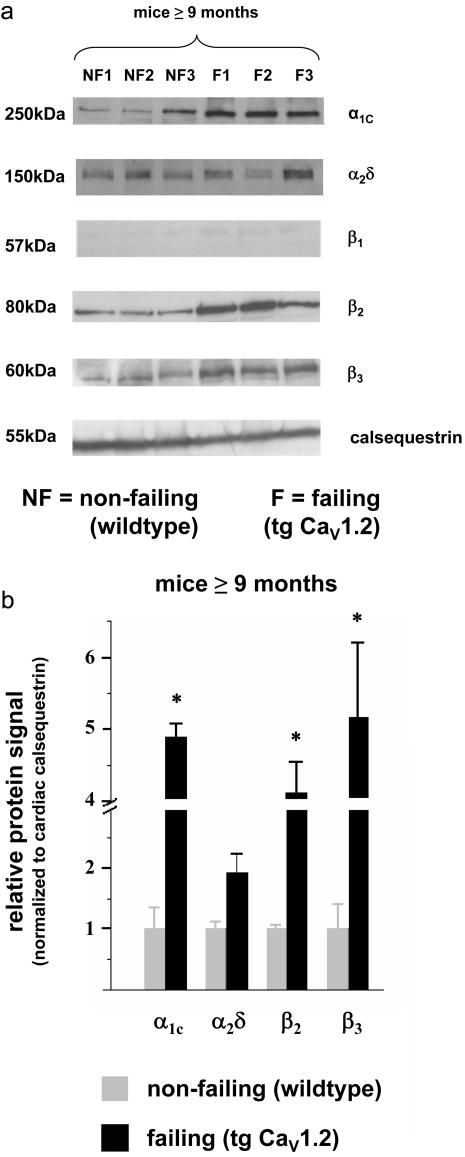
Protein expression of cardiac L-VDCC subunits in old wild-type and tg Ca_V_1.2 mice **(a)** Specimens from old wild-type mice and tg Ca_V_1.2 in heart failure were analyzed in immunoblots using specific polyclonal antibodies directed against the particular L-VDCC subunits. **(b)** Protein expression of L-VDCC subunits was always normalized to cardiac calsequestrin protein expression in the same sample. Quantitative analysis of subunit protein expression is depicted as ratio of 10 months old tg Ca_V_1.2 vs. age-matched wild-type. β_1_ protein bands were faint, and thus not analyzed quantitatively (number of WT/old tg Ca_V_1.2 specimens was always identical for each subunit; n = 4). * p<0.05.

**Figure 3:**
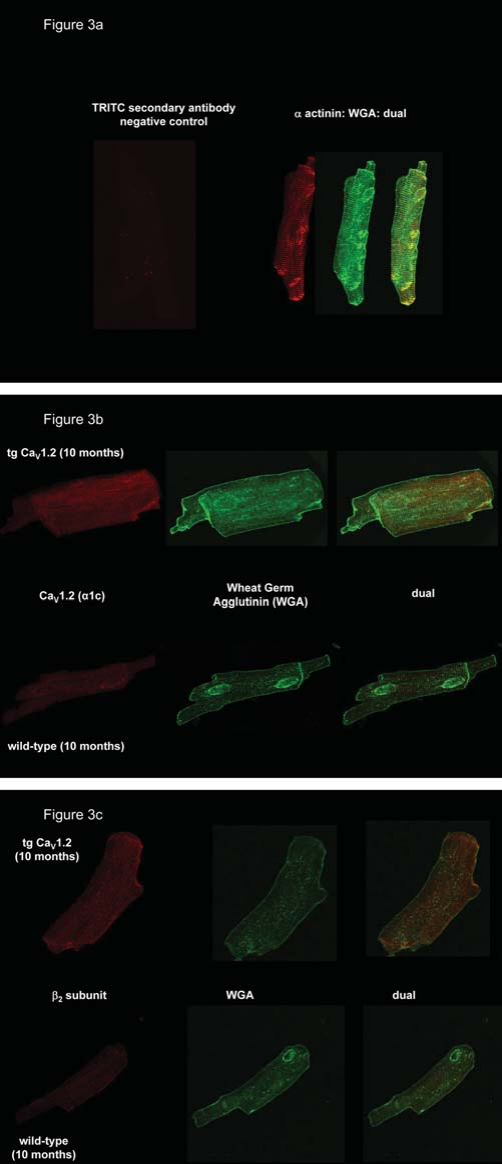
Immunocytochemistry shows increased sarcolemmal expression of Ca_V_1.2 and β_2_-subunits in ventricular myocytes from old tg Ca_V_1.2 mice **(a)** Immunocytochemistry of cardiomyocytes isolated from 10 months old wild-type or tg Ca_V_1.2 mice. Controls show no specific interaction for TRITC labeled secondary antibody and labeling of sarcolemma, T-tubules and intercalated discs by Oregon Green 488-conjugated wheat germ agglutinin (WGA). **(b,c)** Primary antibodies directed against Ca_V_1.2 and β_2_-subunits, respectively, demonstrate predominant t-tubular localization of the respective antigens in old wild-type and tg Ca_V_1.2.

β_1_, β_2_ and β_3_ isoforms are expressed at the protein level in old Ca_V_1.2 mouse heart, although expression of β_1_-subunit isoforms was faint. Compared to old wild type mice (≥9 months), the old tg Ca_V_1.2 showed a significant up-regulation of β_3_-, and β_2_-subunits (Figure 2), in striking contrast to the down-regulation of β_2_-subunits we previously observed in young tg Ca_V_1.2 [16]. In the old tg Ca_V_1.2 mouse myocytes, up-regulated β_2_-subunits and overexpressed Ca_V_1.2 both localize to the surface sarcolemma and the T-tubules (Figure 3), suggesting the functional relevance of altered expression levels.

### β-subunit-dependent modulation of Ca_V_1.2 expressed in HEK293

The diversity of β-subunit expression patterns found in cardiomyocytes necessitated the functional characterization of each β-subunit isoform. Using HEK293 cells constitutively expressing the human Ca_V_1.2 as a homologous recombination system we show that the gene as well as alternative splicing determines calcium channel gating, extending and elaborating on previous work [12]. This is highlighted by significant differences in peak current and open probability between β-subunits (Table 2), with β_2a _and β_2b_ exerting the strongest effects. β_2c_ or β_2d_ as well as β_1a_, β_1c_, and β_3a_ induced a minor to moderate increase in single-channel activity with no significant effects detected for closed times. Taken together, the data support the view that the single-channel phenotype of failing cardiomyocytes is caused by channel complexes containing β_2a_ or β_2b_.

**Table 2:** Gating of single recombinant human Ca_V_1.2 in HEK293 cells is differentially modulated by several coexpressed human cardiac auxiliary β-subunits

gating parameter	β_1a_	β_1c_	β_2a_	β_2b_	β_2c_	β_2d_	β_3a_	control
peak current I_peak_ [fA]	−36±12	−22±5	−109±25*	−123±28*	−24±7	−37±16	−46±14*	−12±2
fraction of active sweeps f_active_ [%]	53.3±10.5	32.5±5.2	57.6±6.6	58.7±9.1	45.5±14.6	64.1±8.2	48.9±6.4	46.9±6.3
open probability P_open _[%]	2.1±0.6	2.4±0.5*	6.9±1.4*	8.3±1.9*	1.8±0.6	3.2±1.3	2.8±0.8(*)	1.1±0.2
mean open time t_open_ [ms]	0.34±0.09	0.28±0.02*	0.31±0.02*	0.34±0.03*	0.23±0.03	0.26±0.01*	0.30±0.03*	0.22±0.01
mean closed time t_closed_ [ms]	11.5±1.7	15.8±5.3	7.0±0.5*	4.7±1.0*	10.8±2.6	11.4±1.7	12.1±2.9	12.4±1.3
number of experiments	10 (6)	11 (4)	14 (6)	16 (8)	5 (4)	9 (6)	10 (8)	8 (5)

Single-channel gating parameters of human cardiac Ca_V_1.2 stably expressed in HEK293 cells transiently cotransfected with several auxiliary β-subunits and a human cardiac α_2_δ-2-subunit. Coexpression with β_2a_ and β_2b_ reveals strongest stimulation of single channels compared to control cells without transfection of any β-subunit (ctr). Data were obtained by patch-clamp recordings using cell-attached configuration (charge carrier: 110mM Ba^2+^; holding potential: −100 mV; test potential: +10 mV for 150 ms). *p<0.05 and ^(^*^)^p = 0.07 in Bonferroni-corrected post-hoc tests versus control, following one-way ANOVA. Numbers of experiments given in parentheses indicate number of experiments with only one channel in the patch.

### Generation of an inducible, heart-specific β_2a_-subunit overexpression mouse (tg_ind_ β_2a_)

**Figure 4:**
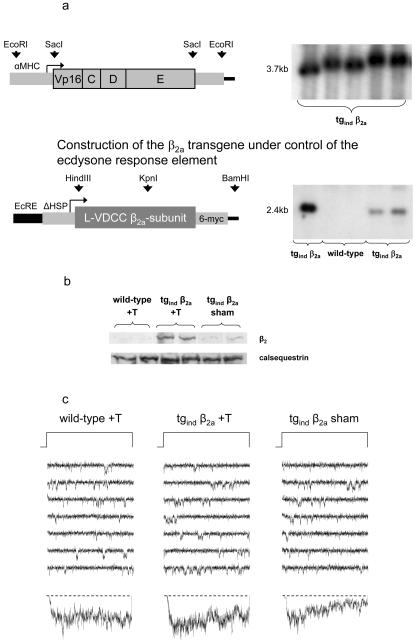
tg mouse model with an inducible cardiac overexpression of the β_2a_ under control of a hybrid bombyx-ecdysone receptor **(a)** For cardiac-specific expression the hybrid bombyx-ecdysone receptor (VgBmEcR) was placed under the control of αMHC promoter (for details see Materials and Methods). Transgenic mice (tg_ind_ β_2a_) positive for the hybrid bombyx-ecdysone receptor and the construct of the ecdysone response element (EcRE) and the β_2a_, respectively, are identified in Southern blots. The radiolabeled probe specific for the coding sequence of VgBmEcR was generated by SacI digestion. It hybridized to a 3.7 kb band in transgene mouse genomic DNA digested by EcoRI digest; the radiolabeled DNA probe specific for the coding sequence of β_2a_ was generated by HindIII/KpnI digest. It hybridized to a 2.4 kb band of genomic DNA digested with HindIII/BamHI in tg_ind_ β_2a_ but not in WT. **(b)** 48 h after treatment with the inducing drug tebufenozide (+T) Western-blot analysis with ventricular tissue from 4–5 month old mice reveals increased expression of β_2_-protein in tg_ind_ β_2a_ compared to treated wild-type or sham-induced transgenics. **(c)** Exemplary traces of single-channel recordings from murine ventricular myocytes. Induction of cardiac overexpression of the β_2a_ (+T) does not alter single-channel behavior compared to either wild-type mice after treatment with the inducing drug or i.p-application of only the vehicle (water/oil-emulsion) to β_2a_-transgenic mice (“sham”). Data were obtained by patch-clamp recordings using cell-attached configuration (charge carrier: 70 mM Ba^2+^; holding potential: −100 mV; test potential: +20 mV for 150 ms). Bottom traces show ensemble average currents from the respective experiment.

**Figure 5:**
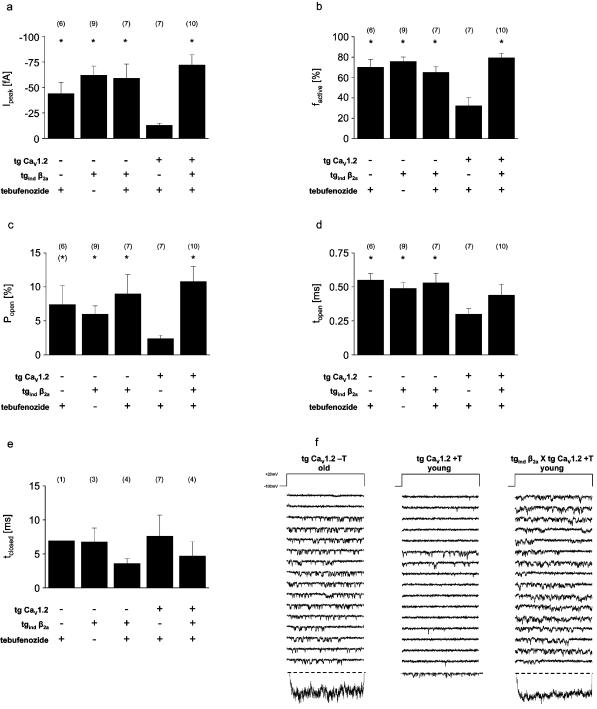
Gating of single L-VDCC in ventricular myocytes from mice showing cardiac overexpression of Ca^2+^-channel subunits **(a–e)** Single-channel gating parameters of ventricular L-VDCC from murine hearts. Compared to 4–5 months old mice showing a cardiac overexpression of the human Ca_V_1.2 (tg Ca_V_1.2), the inducing compound tebufenozide (T) significantly increased single L-VDCC activity in ventricular myocytes from age-matched double-transgenics (tg Ca_v_1.2×tg_ind_ β_2a_, showing an additional inducible β_2a_-overexpression) 48 h after drug administration. Overexpression of the β_2a_-subunit without overexpression of the human Ca_v_1.2 does not alter single-channel gating (cp. Figure 4c). Tebufenozide treatment does not affect single-channel gating in ventricular myocytes from wild-type mice. Data were obtained by patch-clamp recordings using cell-attached configuration (charge carrier: 70 mM Ba^2+^; holding potential: −100 mV; test potential: +20 mV for 150 ms). *p<0.05 and ^(^*^)^p<0.09 compared to tg Ca_v_1.2 in Student's t-test. Number of underlying experiments is given in parentheses. **(f)** Exemplary traces of single-channel recordings from murine ventricular myocytes. Activity of single L-VDCC is clearly higher in old (≥9 months, failing) tg Ca_V_1.2 compared to channels from young (4–5 months, non-failing) tg Ca_V_1.2. Induction of β_2_-overexpression in hearts of young tg_ind_ β_2a_×tg Ca_V_1.2 by tebufenozide mimicks the heart-failure phenotype of L-VDCC gating otherwise not observed until tg Ca_V_1.2 enter the “Maladaptive State” at an age ≥9 months. T = tebufenozide. Data were obtained by patch-clamp recordings using the cell-attached configuration (charge carrier: 70 mM Ba^2+^; holding potential: −100 mV; test potential: +20 mV for 150 ms). Bottom traces show average currents from the respective experiment.

Our functional analyses support the idea of pathophysiological relevance of β_2_-subunit up-regulation, but the parallel biophysical and biochemical changes in cardiomyocytes may still be coincidental. Rather than following the natural course of gene expression changes, transgene-controlled β_2_-subunit overexpression should prove its causative role in native tissue. A hybrid drosophila-bombyx ecdysone receptor (VgBmEcR) when coupled to an αMHC promoter should combine strictly drug-controlled, transgene-specific, and cardiac tissue-specific gene induction. We generated this double transgenic model of αMHC VgBmEcR and the (rat) β_2a_ gene, hence referred to as tg_ind_ β_2a_, under control of the ecdysone response element (Figure 4a). Mice carrying both transgenic constructs developed normally and did not show any signs of developmental or cardiac dysfunction. *In vivo* induction with tebufenozide clearly increased cardiac β_2_-subunit expression in tg_ind_ β_2a_ mice at the protein level (Figure 4b) proofing functionality of drug-controlled gene expression. However, the single L-VDCC phenotype after induction was not altered in this mouse when compared with either tebufenozide treated wild-type mice or sham-induced tg_ind_ β_2a_ (Figure 4c, Figure 5a–e).

### Characterization of L-VDCC activity in double transgenic (tg Ca_V_1.2×tg_ind_ β_2a_) mice

Adaptive down-regulation of β_2_-subunit expression in the young tg Ca_V_1.2 [16] sets the stage for analysis of a β_2_-subunit increase in native tissue. For this we crossbred tg Ca_V_1.2 mice and tg_ind_ β_2a_ mice. Consistent with the hypothesis of a functional predominance of β_1_-subunits in the young tg Ca_V_1.2 mice, (***N.B***.: These animals are “Adaptive”, and in fact demonstrate hypercontractility, without failure) activity of single-channels was dramatically increased by β_2_-subunit induction in tg Ca_V_1.2×tg_ind_ β_2a_ 48 hours after induction (Figure 5) (***e.g.*** peak current: −72±10 fA vs. −13±2 fA in tg Ca_V_1.2 likewise treated with tebufenozide, p<0.05). This is evidence for our proposed relationship of structure and function of L-VDCC, at the single channel level, in ventricular myocytes ***ex vivo***.

## Discussion

The major, novel result of our study is the concomitant increase in β_2_-subunit expression and L-VDCC activity in three independent models: human dilated cardiomyopathy, old tg Ca_V_1.2 mice spontaneously progressing into heart failure and young (“Adaptive State”) tg Ca_V_1.2 mice with additional tissue-specific inducible overexpression of β_2_-subunits. We explain our results by the differential effects of the β-subunits, namely β_2a_ and β_2b_ as the most important modulators in recombinant assays.

The three β-subunits (β_1–3_) thus far known to be expressed in mammalian hearts have vastly different effects on current density [17], [24], kinetics [12], [24], and single-channel properties [12], [14], [17]. Beyond that, some β_2_-subunit isoforms have been implicated in mediating membrane targeting [3], [25], cardiomyocyte apoptosis [26], cell death [17], adrenergic regulation [27], [28] and modulation of CaM Kinase II [29], [30]. It is of interest that the pattern of auxiliary subunits [3], including the prevalence of β-subunit isoforms varies among species, with β_2_-subunits predominating in small rodents like rats [17], and mice [31], and additional relevant expression of β_1_- and β_3_-subunits in humans [13], [19], [23].

In our study the homologous recombination of human L-VDCC subunits indicates the biophysical relevance of β_2a_ and β_2b_ isoforms for up-regulation of single L-VDCC activity. The increase of β_2_-subunit gene expression in human heart failure suggests that these L-VDCC subunits form the basis of the ”heart-failure phenotype” of single L-VDCC found in human hearts [6]. Young tg Ca_V_1.2 animals were chosen as a known example of dynamic adaptation of β_2_-subunit expression in heart. The functional relevance of this adaptation is illustrated by whole-cell current density, that is increased by only 50% [22], [32], while Ca_V_1.2 protein expression and density of single channels show a 3-fold increment in these transgenic hearts [16], [32]. This apparent discrepancy is explained by an up-regulation of β_1_- but substantial down-regulation of the β_2_-subunit expression [16]. This gene expression is characteristic for the “Adaptive phase” of the model [3], [22] putatively limiting calcium overload at the younger ages. We now demonstrate that when this tg Ca_v_1.2 mouse enters into the “Maladaptive state” with overt heart failure at ≥9 months of age, both single L-VDCC activity and β_2_-subunit expression increase, mimicking alterations of channel structure and biophysics in terminal human heart failure. Thus, the old tg Ca_V_1.2 mouse may be regarded as a heart-failure model in which a primary calcium (current) overload can no longer be effectively counterbalanced by adaptive mechanisms, ***i.e.*** β-subunit expression. The transcriptional mechanisms underlying this bidirectional control of β_2_-subunit expression, however, remain to be elucidated in the context of changes in β_1 _and β_3_ subunit expression in human and old tg Ca_V_1.2 mouse heart failure.

As a novel and first approach to induce an increased β_2_-subunit overexpression in intact animals, rather than in isolated cells [17], [24], [26] we generated a mouse model of cardiospecific inducible β_2a_-subunit expression (tg_ind_ β_2a_). Induction of β_2_-overexpression in this mouse model did not affect overall single L-VDCC gating significantly. As a more recent study indicates that an 1:1-stoichiometry of pore-forming α_1_- and auxiliary β-subunit may be sufficient for modulation of channel gating [33], we assume that most calcium channel pores are saturated with native β-subunits in the induced tg_ind_ β_2a_. However, mean closed time was lower in induced tg_ind_ β_2a_ suggesting that a portion of overexpressed β_2a_ exerts functional action similar to the recombinant channel data presented. To prove our concept that β_2_-subunit expression underlies the activity of single L-VDCC of the heart-failure phenotype we crossbred tg_ind_ β_2a_ with tg Ca_V_1.2 mice [21], [22]. Induction of β_2a_-subunit gene expression in the young double tg mice (tg Ca_V_1.2×tg_ind_ β_2a_) led to a premature increase of single L-VDCC activity. This confirms our theory, derived from recombinant channel data, in the relevant tissue ***ex vivo***.

Such deliberate overexpression of β_2_-subunit ***in vivo***, when carried forward in a chronic manner, hopefully will pave the way for understanding the progression of heart failure if these alterations in single L-VDCC gating lead to decompensation at an earlier age of the animal. This knowledge will have direct implications because pharmacological agents which modulate L-VDCC function are in everyday clinical practice and have been shown to be beneficial in various clinical trials targeting different populations [34], [35]. We wish to emphasize that, at this point in our studies, we show a relationship between electrophysiological parameters that is consistent with heart failure. In order to prove this, it is necessary to chronically imbue the young animals with a heart-specific increase in the β_2_ subunit and follow their transition to heart failure at specific age points as they mature. These experiments are ongoing but will require considerable time.

## Materials and Methods

### Materials

Non-failing and failing human left ventricular specimens were obtained from explanted hearts not transplanted for technical reasons (n = 5), or from orthotopic heart transplantation recipients (n = 8). Heart failure patients were in NYHA class III–IV (17–63 y, 3 females) mean duration of symptomatic heart failure ranged from 9–60 months, peak oxygen exercise capacity was 13.3–15.5 ml kg^−1^ min^−1^ at time of listing. Heart transplant recipients were ambulatory at time of operation and received treatment with inhibitors of the angiotensin-system (100%), β-blockers (75%), aldosterone antagonist (50%), diuretics (62.5%).

### Animals

Mice with cardiac-specific heterozygous overexpression of the human Ca_v_1.2 [21] were bred with non transgenic littermates. These animals were bred with the tg_ind_ β_2a_ as described below. Non transgenic littermates served as WT controls in this study.

The study was approved by the local institutional committees.

### Quantitative analysis of L-VDCC subunit mRNA expression in human myocardium

#### Real-time PCR:

Quantitation was performed with the iCycler iQ real-time PCR detection system using primer/fluorescent probe concentrations of 200 nmol/L either in 1× iQ Supermix (β_2a–d_) or 1× iQ SyBr Green when no fluorescent probes was used (β_1a,b_, β_3_, cardiac calsequestrin) (all Bio-Rad®). Both iQ Supermix and SyBr Green based real-time PCR were followed by gel electrophoresis confirming amplification of singular products of expected size. In addition, SyBr Green based real RT-PCR was followed always by melt-curve analysis (70°C–94°C at 0.5°C steps) verifying the similarity of standard and specimen amplification product melting point. Quantitation was performed using intra-assay standard curves of the specific templates. Templates were cloned from human non-failing left ventricle mRNA reversely transcribed by iScript (Biorad®), identity to published sequences was confirmed at both strands.

#### Human β_1_ template:

PCR-cloning by sense primer 5′-CTCAAGGGCTACGAGGTTAC-3′ and antisense primer 5′-GTGTTTGGACTGAGACTTTCC-3′ (GenBank NM_000723; resp. positions: 864-883, and 1247-1227), with 94°C (3 min), and 40 cycles of 51°C (30 sec), 72°C (20 sec), and 94°C (20 sec). Real-time PCR was set up with the cloning primers and run at 94°C (3 min), and 40 cycles of 50°C (30 sec), 72°C (20 sec), and 94°C (20 sec). Correlation: ≥0.992, efficiency: ≥93.4%.

#### Human β_1_ isoform templates:

PCR cloning of the β_1a_ by sense primer 5′-GCCTCGGCTCCAGCAAA-3′ and antisense primer 5′-CTCACCAAGCTCAGCCTCTTC-3′ (GenBank NM_199247; resp. positions 692-708; 855-835), of the β_1c_ by the same sense but different antisense primer 5′-CTCTGTCGACTTCTGCTTCTGTTT-3′ (GenBank NM_000723; position 807-784), with 94°C 3 min, 40 cycles 56°C (30 sec), 72°C (20 sec), 94°C (20 sec). Real-time PCR was set up with the respective cloning sense primer and antisense primers, and the fluorescent probe 5′-6FAM-CTCCAGTTCCAGTCTGGGAGATGTGGT XT p (GenBank NM_000723; 720-746) and run at 94°C (3 min), and 40 cycles of 53°C, and 94°C (each 30 sec). Correlation: ≥0.990, efficiency: ≥90.6%.

#### Human β_2a–d_ isoform templates:

PCR cloning of β_2a–d_ isoforms was by isoform specific sense primers:

β_2a_: 5′-GCATCGCCGGCGAGTA-3′, (GenBank423189, position: 21-36);

β_2b_: 5′-GACAGACGCCTTATAGCTCCTCAA-3′ (GenBank AF285239; position: 7-30);

β_2c_: 5′-AGTGGACTGGACCTGCTGAA-3′ (GenBank AF423190; position: 13-32);

β_2d_: 5′-GCCGCCGCACAGTCATAT-3′ (GenBank AF423191; position: 109-126);

always with the same antisense primer 5′-CGGTCCTCCTCCAGAGATACAT-3′ (GenBank AF423189; position: 109-89). Real-time PCR was set up with the respective cloning primers, and the fluorescent probe 5′-6FAM-ATGGACGGCTAGTGTAGGAGTCTGCCGA XT p (GenBank NM_000723; position: 79-52) and run at 94°C (3 min), and 40 cycles of 56.5°C, and 94°C (each 30 sec). Correlation was ≥0.995, efficiency ≥90.4%.

#### Template of human β_3_ and human cardiac calsequestrin:

see [12].

### Cloning of human β_1_-, β_2_-_, _β_3_-splice variants and insertion into bicistronic eukaryotic expression vectors

#### β_1_-subunits:

Full length β_1_-subunit isoform sequences were cloned using two pairs of sequence specific primers: 1^st^ sense primer 5′-CCTCTCCATGGTCCAGAAGACCAGCA-3′ and 1^st^ antisense primer 5′-CAAATAAAGCTTTCTGCATCATGTCTGTAA-3′, and 2^nd^ sense primer 5-TTACAGACATGATGCAGAAAGCTTTATTTG-3′ and 2^nd^ anti-sense primer 5′-GCGCCCACTACATGGCATGTTCCT-3′ (GenBank NM_199247; resp. positions: 147-173; 1048-1019; 1019-1048; 1732-1709). PCR with the 1^st^ primer pair yielded two amplification products due to alternative splicing of exon 7a and exon 7b. Full length message of β_1A_ with either exon 7a or 7b was reassembled in pIRES2-EGFP opened with *Bgl*II/*Sma*I site using the internal *Hind*III restriction site.

#### β_2_-subunits:

Full length β_2_-subunit isoform sequences were cloned using two pairs of sequence specific primers derived from GenBank sequences. N-terminal coding sequences for β_2_-subunit splice variants were generated using isoform specific primer pairs. β_2a_: sense primer 5′-CTCTTCATGCAGTGCTGCGGGCTGGT-3′ and antisense primer 5′- ACTTCCGCTAAGCTTGACCTTGTG-3′ (GenBank U95019; resp. positions: 496-521, 1397-1374); β_2b_: sense primer 5′-ATGCTTGACAGACGCCTTATAGCT-3′ and same antisense primer (GenBank AF285239; resp. positions: 1-24, 899-876); β_2c_: sense primer 5′-ATGAATCAGGGGAGTGGACTGGAC-3′ and same antisense primer (GenBank AF423190; resp. positions: 1-24, 977-894); β_2d_: sense primer 5-ATGGTCCAAAGGGACATGTCCAAG-3′ and same antisense primer (GenBank AF423191; resp. positions: 1-24, 1061-1038). The C-terminal fragment was amplified by sense primer 5′-CACAAGGTCAAGCTTAGCGGAAGT-3′ and antisense primer 5′-GGCAAAACTCATTGGGGGAT-3′ (GenBank U95019; resp. positions: 1374-1397, 2327-2308). Amplification was performed in cDNA reverse transcribed (Revert Aid Kit, MBI Fermentas®) from mRNA isolated from non-failing human left-ventricular myocardium using Trizol (Invitrogen®) and the poly(A)tract kit (Promega®). PCR conditions always were 40 cycles of 94°C, 58°C, 72°C, (each 1 min); and 5 min 72°C. Amplification products were visualized by UV protected 0.8% agarose gel-electrophoresis, extracted (Perfect Gel Clean-up, Eppendorf-Vaudaux®), and subcloned into pCR2.1-TOPO (Invitrogen®). Sequences of cloned fragments were determined on both strands (MWG-Biotech®). For eukaryotic expression full length β_2_-subunit isoforms were reassembled in the pcDNA3 polylinker region (Invitrogen®) opened by *Bam*HI/*Not*I using the internal *Hind*III restriction site. Full length coding sequences were inserted by T4 DNA ligation of N-terminal β_2_-subunit isoform fragments cut by *Bam*HI (pCR2.1 restriction site) and *Hind*III (internal restriction site contained in all β_2_-subunit isoforms) and of the C-terminal fragment cut by *Hind*III and *Not*I (pCR2.1 restriction site). Full-length coding sequences of β_2_ isoforms were inserted by T4 DNA ligation into pIRES2-EGFP opened by *Eco*RI restriction.

#### β_3_-subunits:

Full length coding sequence was excised by *Eco*RI/*Xho*I and inserted into pIRES2-dsRed2 opened with *Eco*RI/*Sma*I.

#### human α_2_δ-2:

Full length coding sequence was obtained from Klugbauer *et al*. [36] excised by restriction with *Hind*III/*Xho*I and inserted into pIRES2-dsRed2 opened with *Nhe*I blunt/*Xho*I.

### Western-blot analysis of Ca^2+^-channel subunits

Protein expression levels of the L-VDCC subunits were assayed by Western-blot analysis of human and mouse cardiac ventricular protein samples. Briefly, protein extracts were obtained by homogenizing frozen heart tissue in buffer (5% SDS, 50 mM TRIS-HCl, pH 7.4, 250 mM sucrose, 75 mM urea, and 10 mM DTT containing complete protease inhibitor cocktail tablet from Roche) using a Teflon homogenizer. The homogenate was denatured by incubation at 95°C (2 min) followed by centrifugation at 16,000g (5 min); supernatants (containing membrane fractions and cytosolic proteins) were collected for analysis. Protein was quantified using Bicinchoninic acid (BCA) Protein Assay (Pierce®). For Ca_V_1.2 and α_2_δ-1 Western blots, 60 µg, and for β_2_ and β_3_ Western blots, 150 µg of total protein were separated on a 8% and 12% SDS-PAGE gel (BioRad®). Gels were transferred to nitrocellulose membranes (Amersham®) according to standard wet transfer procedure. L-VDCC subunits were detected using the following antibodies: anti-human Ca_V_1.2 against the II-III loop (generous gift from Dr. Hannelore Haase, Max-Delbrück Center, Berlin, Germany; [37], [38]); anti-β_2_ (generous gift from Dr. Adolfo Cuadra (Dr. M. Hosey), Northwestern University, Chicago, USA; [39]); anti-β_3_ and anti-α_2_δ−1 (Alomone;[40], [41]), and anti-calsequestrin (Santa Cruz;[42]). The anti-β_1_ (Swant; cp. [16]) stains a band at ∼57 kDa in the membrane of human skeletal muscle (data not shown) where β_1a_ is pre-dominant [43] suggesting that the antibody detects a β_1a_ in human and mouse myocardium. In the present study this antibody detected an additional band of ∼65 kDa in murine myocardium and ∼70 kDa in human heart. Though our present mRNA data indicate that there are two β_1_-isoforms in cardiac tissue we cannot exclude a cross-reaction of the antibody with β_3_-subunits since the second band is quite close to the band detected by the β_3_-specific antibody from Alomone (see above). Thus we decided to avoid any quantitation of this “high molecular band” detected in murine and human cardiac tissue, respectively.

### Immunofluorescence analysis of Ca^2+^-channel subunits

Ventricular myocytes were freshly isolated from 10 month old tg Ca_V_1.2 and controls as previously described, stored in Kraft-Bruehe solution and plated on laminin-coated with poly-L-lysine and 50 µg/ml mouse laminin (Invitrogen®) coverslips for 1 h at 37° C, 5% CO_2_. After incubation myocytes were washed with relaxation buffer (mM: 100 KCl, 5 EGTA, 5 MgCl_2_, 0.25 dithiothreitol (DTT) in PBS, pH 6.8). Myocytes were then fixed in pre-cooled (−20°C) methanol/acetone (1∶1) for 5–10 min at 4°C. To prevent non-specific binding, myocytes were blocked with 10% normal donkey serum (Sigma®) in PBS overnight (labeling buffer) [44]. Primary antibodies were diluted in labeling buffer and incubated with myocytes overnight at 4°C. Primary antibody dilutions for different subunits of the L-VDCC studied were: 1∶200 for α_1C_ (Alomone®) and 1∶500 for β_2_. In the case of Wheat Germ Agglutinin (WGA) labeling, myocytes were incubated overnight at 4°C with Oregon Green 488-conjugated WGA (Molecular Probes®) at a concentration of 1 µg/ml. WGA selectively binding to N-acetyl-d-glucosamine in glycoproteins was used to label the peripheral sarcolemma, the T-tubules and the intercalated disks. After overnight incubation, myocytes were washed with PBS and incubated with secondary antibody in PBS-0.1% BSA for 1 h at room temperature. Secondary antibody for the study of the L-VDCC subunits was tetramethylrhodamine-isothiocyanate (TRITC)-conjugated donkey anti rabbit antibody at 1∶400 dilution (Jackson ImmunoResearch®, USA). For negative control experiments, myocytes were kept in labeling buffer overnight without primary antibody and only incubated with secondary antibody at the same concentration. After washing the cells with PBS, coverslips were mounted on slides using Gel/Mount aqueous mounting media (Biomeda®) and images were acquired on a Nikon PCM 2000 laser confocal scanning microscope as 0.5 mm “optical sections” of the stained cells, keeping gain and background values constant through the different samples.

### Generation of transgenic mice with inducible cardiac overexpression of β_2a_

Recent modifications in the Drosophila ecdysone receptor revealed better regulation of gene expression in mammalian cells, however, the dependence on steroidal ligand activation (i.e. ponasteron) with its potential additional effects on gene expression remains. The ecdysone receptor from Bombyx mori is activated by the non-steroidal ligand tebufenozide (effective drug in MIMIC, Dow AgroSciences®, Munich, Germany) without known specific interaction in mammalian cells. This construct regulated β-galactosidase expression in HEK293 cells at concentrations of 1 µM tebufenozide as effectively as the Drosophila ecdysone receptor (data not shown). For our experiments we intraperitoneally injected 9 mg tebufenozide (i.e. twice the dose leading to maximum serum concentration of the drug) 48h before isolation of cardiac myocytes. The hybrid drosophila-bombyx ecdysone receptor (VgBmEcR) was constructed by fusion of the binding and transactivation domain of the modified drosophila system (pVgRXR, Invitrogen®) to the ligand binding domain of the bombyx ecdysone receptor (BmEcR in pBSII KS+BmB1 = ecdysone receptor type B1 of Bombyx mori, obtained from Fujiwara H., Tokyo, Japan) using the restriction enzyme *BsrG*I and *Not*I. The coding sequence of VgBmEcR was set under control of the promoter of αMHC for cardio-specific expression. The αMHC promoter was excised from pBlue-MHCβ_1_ARSV40polyA (obtained from Stefan Engelhardt, Wuerzburg, Germany) using *Dra*I and *Pvu*II and inserted into pVgBmEcR opened with the same enzymes. The ecdysone-regulated plasmid pInd-β_2a_ was constructed by excision of the β-galactosidase coding sequence from pInd-LacZ (Invitrogen®) using *Hind*III and *Xba*I and insertion of the coding sequence for rat β_2a_ excised from pCR3-β_2a_-6myc (obtained from A.J. Chien, Chicago, USA) with the same restriction enzymes. The linearized coding sequence of constructs were injected simultaneously into embryonic stem cells, and mice transgenic for the VgBmEcR and the β_2a_-subunit were identified by PCR using construct-specific primers (not depicted), and by Southern blot using αMHC-VgBmEcR and β_2a_ specific probes labeled radioactively. Mouse DNA was obtained from mice 3 weeks post delivery and digested with *EcoR*I for proof of αMHC-VgBmEcR-, and *Hind*III/*BamH*I for Ind-β_2a_ -genomic integration. Probes specific for αMHC-VgBmEcR and Ind-β_2a _were obtained by *Sac*I and *Kpn*I/*Hind*III restriction of the respective coding sequences. Probes were radio-labeled with α-^32^P-CTP using the Klenow fragment. Animals positive for integrated coding sequences were identified by 3.7 kb hybridization signal for αMHC-VgBmEcR and a 2.4 kb signal for Ind-β_2a_ (Figure 4a).

### Cell culture and co-transfection

Cell culture and transient co-transfection were done as described [12], [45]. In brief, HEK293 cells were stably transfected with the full-length Ca_V_1.2-subunit (GenBank NM_000719) cloned from human heart [46]. Cells were seeded in polystyrene Petri dishes (9.6 cm^2^, Falcon®, Heidelberg, Germany) at a density of 1–2⋅10^4^ cells cm^−2^ and transiently co-transfected with the cDNA plasmids encoding the different human cardiac β_2_ splice variants together with human cardiac α_2_δ-2-subunit [36]. Lipofection was carried out 24–36 h after plating by incubating (3–6 h) with SuperFect (Qiagen®) and the respective plasmids at a DNA mass ratio of 3∶3∶1 [12], [45]. Transfected cells were grown on Petri dishes in Dulbecco′s modified Eagle's medium (DMEM, Biochrom KG®, Berlin, Germany) supplemented with 10% fetal bovine serum (Sigma®, Deisenhofen, Germany), penicillin (10 units ml^−1^) and streptomycin (10 µg ml^−1^, both from Biochrom®). Electrophysiological recordings in GFP-positive cells were obtained 48–72 h after transfection.

### Isolation of ventricular myocytes

Single ventricular myocytes were isolated from murine hearts by enzymatic dissociation using the method described earlier [47]. In brief, hearts were perfused with a collagenase solution (Worthington type I and II, 75 U l^−1^) in a Langendorff setup and subsequently cut into small chunks. Myocytes were harvested by pouring the suspension through cheesecloth.

### Single-channel recording

Single-channel recordings were performed by using the cell-attached configuration of the patch-clamp method as described earlier [47]. Cells were placed in disposable Petri dishes containing 3 ml of a high-potassium depolarizing solution (mM: 25 KCl, 120 K-glutamate, 2 MgCl_2_, 10 Hepes, 2 EGTA, 10 dextrose, 1 CaCl_2_, 1 Na_2_-ATP; pH 7.3 with KOH). Patch pipettes (borosilicate glass, 6–8M) were filled with pipette solution for myocytes (mM): 70 BaCl_2_, 110 sucrose and 10 Hepes; for HEK cells (mM): 110 BaCl_2_, 10 Hepes; pH 7.4 with TEA-OH. Ba^2+^ currents were elicited by voltage steps (150 ms at 1.66 Hz) from −100 mV to +20 mV (native channels) or +10 mV (recombinant channels) (≥180 sweeps per experiment). Data were sampled at 10 kHz and filtered at 2 kHz (3 dB, four-pole Bessel) by using an Axopatch 200A amplifier (Axon Instruments®, Foster City, USA). PCLAMP software (CLAMPEX 5.5.1, FETCHAN, and PSTAT 6) was used for data acquisition and analysis (Axon Instruments®, Foster City, USA). Signal-noise ratio and adequate resolution of openings were similar to previous work [12], [16], [47], as confirmed by comparison of, ***e.g.*** data from wild-type mice.

### Data analysis and statistics of single-channel recordings

Linear leak and capacity currents (averaged non-active sweeps) were digitally subtracted. Openings and closures were identified by the half-height criterion. The fraction of active sweeps within a patch (f_active_), the open probability within active sweeps (P_open_), and the peak value of single-channel ensemble average currents (I_peak_) were determined as described [16]. Where necessary, these parameters were corrected for the number of channels in a patch, as described [6]. For comparisons unpaired Student's two-tailed t-test or Mann-Whitney test was used where appropriate. Throughout, a level of p<0.05 was considered significant. Values are given as mean±SEM.
